# Strategies to Mitigate a *Mycobacterium marinum* Outbreak in a Zebrafish Research Facility

**DOI:** 10.1089/zeb.2015.1218

**Published:** 2016-07-01

**Authors:** Timothy Mason, Kathy Snell, Erika Mittge, Ellie Melancon, Rebecca Montgomery, Marcie McFadden, Javier Camoriano, Michael L. Kent, Christopher M. Whipps, Judy Peirce

**Affiliations:** ^1^ Aquatic Animal Care Services, University of Oregon, Eugene, Oregon.; ^2^ Institute of Molecular Biology, University of Oregon, Eugene, Oregon.; ^3^ Institute of Neuroscience, University of Oregon, Eugene, Oregon.; ^4^ Department of Microbiology and Biomedical Sciences, Oregon State University, Corvallis, Oregon.; ^5^ SUNY-ESF, State University of New York College of Environmental Science and Forestry, Syracuse, New York.

## Abstract

In 2011, the zebrafish research facility at the University of Oregon experienced an outbreak of *Mycobacterium marinum* that affected both research fish and facility staff. A thorough review of risks to personnel, the zebrafish veterinary care program, and zebrafish husbandry procedures at the research facility followed. In the years since 2011, changes have been implemented throughout the research facility to protect the personnel, the fish colony, and ultimately the continued success of the zebrafish model research program. In this study, we present the history of the outbreak, the changes we implemented, and recommendations to mitigate pathogen outbreaks in zebrafish research facilities.

## Introduction

Many species of mycobacteria are associated with mycobacterial infections of zebrafish^1,2^ and most are considered opportunistic pathogens.^1^ Two species, however, *Mycobacterium haemophilum* and *Mycobacterium marinum*, are seen as more virulent based on laboratory transmission studies and mortalities associated with natural infections^1–4^ and therefore pose a greater risk to research programs utilizing the laboratory zebrafish model. Outbreaks of *M. haemophilum* in laboratory zebrafish colonies have been described as severe and persistent^2^ and although outbreaks of *M. marinum* have not been similarly documented in zebrafish facilities, there is a sufficiently recognized association between increased zebrafish mortalities and *M. marinum* infection to group *M. marinum* and *M. haemophilum* as pathogens of greatest concern^1^ to laboratory zebrafish colonies.

In this study, we describe an outbreak of *M. marinum* that affected a large colony of zebrafish (*Danio rerio*) in an indoor vivarium dedicated to zebrafish research. Zebrafish observed with ulcerative lesions were sent for diagnostic pathology testing that ultimately revealed infections caused by *M. marinum*. At the same time, a facility staff person discovered a hand infection that medical professionals determined was caused by *M. marinum*. Large-scale depopulation and facility disinfection were not possible given the need to continue sponsored research and support facility users, so instead we implemented a plan for managing and reducing the impact of the pathogenic mycobacterium.

In this report, we detail changes made to the personnel management, facility husbandry, and veterinary care, designed to minimize the impact of the *M. marinum* outbreak, and we discuss the effectiveness of the management plan.

## Facility Overview

All animals described in this report were housed in the Aquatic Animal Care Services Zebrafish Facility on the campus of the University of Oregon. The University of Oregon Animal Care Program is accredited by AAALAC International and the zebrafish research and husbandry procedures described here were approved by the University of Oregon Institutional Animal Care and Use Committee.

### Water system

Our facility utilized a recirculating aquaculture system (RAS) comprising mechanical filters, biological filtration, and ultraviolet disinfection for its life support system. One large RAS made of two linked, but separable, systems was used for all the fish in the colony. We replaced water lost from evaporation, spills, fish husbandry procedures, and nitrate removal processes using water obtained through reverse osmosis filtration. We added sea salts, bicarbonate, and crushed coral (calcium carbonate) to the bioreactors through dosing pump stations as our water quality meters demanded. Our system used air blowers to provide particulate-filtered room air to facilitate water oxygenation, carbon dioxide degassing, and bioreactor media movement.

### Quarantine

Biosecurity for our facility was partly maintained through the use of a strict quarantine practice that mandated only embryos that had been surface disinfected with sodium hypochlorite could enter the colony. No live adult zebrafish from outside the colony was allowed into it. Live imported zebrafish larvae, juveniles, and adults were housed in our quarantine facility in the same building as the main colony, but separated by several building floors. The quarantine facility used a flow-through (single-pass) life support system to minimize pathogen spread among imported fish.

### Feed

We fed our zebrafish different feed types as the fish grew to different developmental stages. We fed zebrafish larvae live rotifers (*Brachionus plicatilis* L-type; Reed Mariculture, Campbell, CA) in a solution of slightly brackish water (5 parts per thousand salt) with microalgae (Rotigrow Plus; Reed Mariculture). We fed older larvae [(>10 days postfertilization (dpf)] and juvenile (30–60 dpf) zebrafish live brine shrimp nauplii (*Artemia franciscana,* Artemia International, LLC, Fairview, TX). We fed our adult and juvenile zebrafish a dry crumble commercially manufactured feed (New Life International, Inc., Homestead, FL). Our facility staff and student workers manually delivered feed to each tank.

## Case History, Diagnostics, Treatments, and Outcomes

### History and diagnostics

In late 2010 and early 2011, we noticed an increased number of zebrafish in the facility with ulcerative lesions on their skin. We sampled some fish and sent them to the Zebrafish International Resource Center (ZIRC) Health Services (http://zebrafish.org/health/) for diagnostic testing through histopathology. Bacterial cultures isolated from sampled fish were highly suspect for *M. marinum* as they produced yellow colonies after about 1 week of culture on blood agar. DNA sequence of the *hsp65* gene confirmed that the pathogen causing the mycobacteriosis was *M. marinum*.

At the same time, in the spring of 2011, a member of the zebrafish research facility staff became aware of a red sore on his hand that did not appear to be healing. This individual began to investigate his hand issue and because all UO zebrafish researchers and staff receive information about zoonotic infection, he was aware of the possibility that his affliction could be caused by a zoonotic fish pathogen. After several visits to physicians, testing through PCR revealed that the hand infection was caused by *M. marinum*.

In total, three persons working in the facility discovered reddish bumps on one hand, suggesting an *M. marinum* infection, although only one of these was confirmed. Despite indications pointing to the conclusion that the *M. marinum* pathogen that caused the zoonotic infection in the staff member was the same pathogen causing infections in the colony zebrafish, no specific testing, for example, DNA comparison, was performed to confirm this assumption.

### Treatments and outcomes

Treatments for personnel were directed by medical professionals. We did not attempt to treat any infected zebrafish. We culled colony zebrafish that exhibited clinical signs, including ulcerative lesions and exophthalmia.

Our health monitoring program before the outbreak had consisted primarily of biannual sentinel fish sampling with an occasional case due to concerns over clinical signs of disease, either physical (color change, weight loss, distended abdomen, ulcerative lesion, exophalmia, etc.) or behavioral (lethargy, twirling, etc). Fish from sentinel tanks placed alongside other housing tanks on racks as well as fish from sentinel tanks receiving effluent water from all other facility fish were evaluated using histopathology by the ZIRC Health Services. Rarely, a single zebrafish or small group of zebrafish, showing clinical signs of disease, was similarly sent to the ZIRC Health Services for diagnostic evaluation through histopathology. During the outbreak and continuing afterward, we increased our sentinel sampling frequency to quarterly testing and we began to use environmental sampling.

To assess the prevalence and location of *M. marinum* in the facility itself, we implemented a program of weekly environmental sampling. Environmental sampling as a supplement to sentinel animal programs has been used in rodent facilities.^5^ Sample sites were randomly chosen and included biobeds, feed, water hoses, equipment, and supply cabinets from various rooms in the facility (Table 1). In the beginning of the program, samples were taken to a laboratory specializing in bacteriology at the University of Oregon for testing through PCR and bacterial culture. Later, samples were sent to a commercial bioresearch laboratory (IDEXX BioResearch, Columbia, MO) for testing through PCR.

**Table 1.** Summary of Environmental Sampling for Prevalence

*Sample site*	*Mycobacterium* spp.
Biobed	+
Buffer tanks	+
Computer keyboard in fish housing room	+
Embryo collection egg strainer	−
Euthanasia chamber: interior	+
Euthanasia chamber: top and handle	−
Floor: dirty side washroom	−
Floor: facility entrance PPE station	−
Floor: under rotifer cultures	+
Footwear: bottom of facility-provided plastic dedicated footwear (PPE)	−
Handle on door to clean equipment cabinet	+
Hose attached to embryo medium carboy: water outlet end	−
Hose attached to RO unit: water outlet end	−
Hose for filling breeding cages: biofilm from cut cross section	+
Hose for filling breeding cages: water outlet end	+
Rotifers from vendor	−
Rotifers from laboratory culture	+
Rotifer culture bucket biofilm	+
Rotifer strainer	−
Microscope	−
Water inlet valve for housing tank	+

Summary data from sites sampled over a 6-month period from June 2013 to January 2014. *Mycobacterium* species presence was tested using PCR assays by a University of Oregon laboratory specializing in bacteriology. + indicates that this area was positive for mycobacteria at least once.

− indicates that all samples from this area were negative for mycobacteria.

To assess the prevalence of *M. marinum* in our zebrafish population, we updated our sampling program to include zebrafish with signs of disease and continued to sample sentinel zebrafish quarterly. Fish sampled were either fixed or frozen. Fixed fish were reviewed through histopathology, followed by PCR testing of suspect fixed tissues in the paraffin block (Oregon Veterinary Diagnostic Laboratory, Corvallis, OR). Frozen fish were sent for diagnostics and PCR testing (IDEXX BioResearch, Columbia, MO) (Table 2).

**Table 2.** Histopathology Results Related To Mycobacteria

	*Year 2007*	*Year 2008*	*Year 2009*	*Year 2010*	*Year 2011*	*Year 2012*	*Year 2013*	*Year 2014*	*Year 2015*
Sampled fish count	19	36	97	44	290	72	150	346	134
Sampled fish having acid-fast bacilli (AFB)^a^	0	6	13	10	31	9	7	13	9
AFB caused by *Mycobacterium chelonae*	0	5^b^, 1^c^	5^b^, 2^c^	5^b^, 1^c^	3^b^, 2^c^	1^c^	1^c^	3^b^, 5^c^	2^b^, 1^c^
AFB caused by *Mycobacterium fortuitum*	—^d^	—^d^	—^d^	—^d^	—^d^	—^d^	—^d^	—^d^	1^e^
AFB caused by *Mycobacterium haemophilum*	0	0	0	1^c^	0	0	0	0	0
AFB caused by *Mycobacterium marinum*	0	0	1^c^	0	2^b^, 2^c^	0	0	1^b^	1^c^
No test performed^f^	0	0	1	3	7	4	6	1	—^**h**^

For reasons described in the text, the numbers in this table from 2007 to 2011 may represent an underestimate of the number of affected fish, whereas sampling since 2012 has been more systematic and thus more accurate.

^a^ Ziehl-Neelsen staining revealed acid-fast bacilli.

^b^ Fixed fish from paraffin block found to be positive (Ct = 30–38) using qPCR.

^c^ Fixed fish from paraffin block found to be weak positive (Ct > 38) using qPCR.

^d^ Test for *M. fortuitum* not performed.

^e^ Test for *M. fortuitum* performed on fresh or frozen tissue.

^f^ Retrospective qPCR test not performed on these individuals.

^h^ No retrospective testing performed after January 2015.

We implemented a number of changes to personnel management, husbandry, and veterinary care in an effort to better track disease and to eliminate the existence and the spread of the pathogenic mycobacteria. These strategies (Table 3) evolved quickly because of the concerns for the ongoing research projects and the perception that *M. marinum* was rapidly spreading through our colony based on the increased incidence of fish seemingly in poor health.

**Table 3.** Strategies to Mitigate Issues from a Mycobacterial Outbreak

*Strategy*	*Rationale*	*Practical effect*
Provide personnel training on mycobacteria	*Guide for the Care and Use of Laboratory Animals,* 2011	Reduces risk of zoonotic infection; Reduces pathogen vectors
Wear personal protective equipment (gloves)	*Guide for the Care and Use of Laboratory Animals,* 2011	Reduces risk of zoonotic infection
Use 70% ethanol to disinfect facility surfaces and hands	Mainous 2005	Eliminates bacteria on facility surfaces and hands
Use embryo surface disinfection	Our experimental results	Reduces bacterial counts on embryo chorion
Track diseased fish with tank labels	*Guide for the Care and Use of Laboratory Animals,* 2011, and observations at UO	Provides surveillance data
Perform environmental sampling	Adapted from rodent health monitoring. Pritchett-Corning 2014.	Provides surveillance data
Plan and direct personnel movements	*Guide for the Care and Use of Laboratory Animals,* 2011	Reduces pathogen spread through personnel movements
Remove elderly fish	Keller 2004 and Sasaki 2013	Removes potential disease carriers
Remove dead and moribund fish	Kent 2009	Removes potential disease carriers
Place young fish highest on housing racks	“Because water is an excellent vehicle for pathogens.” Kent 2009	Reduces risk of pathogen spread through water spill
Dedicate wild-type fish for outcrosses	Noga 2010 and Murray 2012	Reduces pathogen spread through shared fish for outcrosses
Remove spawn water and water from tank changes from RAS	Adapted from Murray 2012	Eliminates potentially pathogenic bacteria from RAS
Change tanks every 3 weeks	Observations in our facility	Reduces biofilm and algae
Evaluate and validate sanitation	*Guide for the Care and Use of Laboratory Animals,* 2011	Reduces pathogen spread through soiled equipment

There was no central recordkeeping system for zebrafish culled as the result of *M. marinum* infection signs because individual laboratories maintained research strains and population management records themselves. Therefore, although we present data about the results of sampling our zebrafish (Table 2), these are probably not an accurate reflection of *M. marinum* prevalence within our colony at the early stages of the outbreak in 2010 and 2011. We centralized our sampling in 2012, thus numbers presented beginning in that year are an accurate representation of disease prevalence.

Because of the seemingly poor condition of many fish starting in 2010, there was a widely understood urgency to remove diseased zebrafish from our colony immediately upon their discovery. Because of our urgency, some of our strategies came from our observations and suppositions—sometimes supported by scientific literature—and others came from experimental results.

#### Personnel management

Through the help of the campus biosafety officer in the Office of Environmental Health and Safety (EHS), we implemented changes to protect people having direct contact with colony zebrafish. Changes to protect personnel consisted of updated training information with an emphasis on zoonotic infection, including signs of infection such as reddish bumps (papules) that enlarge over time, along with swollen lymph node, and the mandatory use of personal protective equipment (PPE), especially hand protection through the use of gloves.

EHS required us to post a sign at the entrance to the fish housing rooms that indicated a potential zoonotic pathogen was present and that gloves were required. Previous to this outbreak, we did not require PPE and most personnel used no gloves for work involving direct contact with our zebrafish. Our regular facility personnel requested a glove with a long cuff to minimize the chance of water entering the glove at the cuff.

Materials, including gloves, which contact laboratory zebrafish systems, can leach potentially toxic materials into the water and should be tested before use.^6^ Our material toxicity testing revealed that a particular nitrile glove with long cuff (TechNitrile TN1200 Series Class10, 12″) was nontoxic to the zebrafish and we therefore chose this glove. We created a clear separation between personnel space and animal space and used a single point of entry to the zebrafish housing and procedure rooms to establish a PPE station. We mandated that personnel in the zebrafish housing and procedure spaces must wear PPE provided by the facility, including gloves, dedicated shoes (or disposable shoe covers), and long pants. In addition to mandatory glove use, we provided 70% ethanol solution at all work stations to disinfect countertops, carts, egg collection strainers, small tank aspirators, and gloved hands because 70% ethanol has been shown to be effective against *M. marinum.*^7^

The use of PPE in the facility was mandated by our EHS and therefore any person found in the facility without the proper PPE could be removed from it and retrained on the risks of zoonotic infection. In our experience, however, no personnel refused to wear the PPE. Importantly, infections affecting personnel were reduced from three cases in 2011 (1 confirmed, 2 presumed based on clinical signs) to zero cases in all the years following.

Our environmental sampling results not only revealed the presence of mycobacteria in areas with fish water, for example, biobeds and hoses used to fill breeding cages, but also showed examples of personnel contaminating facility equipment (Table 1). We found positive results for mycobacteria on cabinet handles and a computer keyboard, although negative results were found on egg strainers and microscope knobs. The contaminated surfaces in the facility showed us how easily we could spread the mycobacteria to any place in our facility, even with the updated training.

To prevent further spread of the mycobacteria by personnel, we showed our test results to staff and researchers and then provided more wash bottles containing ethanol in all areas of the facility and encouraged all personnel to either disinfect their gloves regularly using 70% ethanol or to change their gloves regularly. We also restricted specific areas such as the food preparation area to trained staff only in an effort to prevent spread of the pathogen.

#### Husbandry and veterinary care

Because fish strains from outside sources were only permitted into the facility through disinfected embryos, we suspected that the quarantine and disinfection procedures we used were inadequate and ineffective at preventing *M. marinum* from entering the colony. Our previous disinfection consisted of two 5-min immersions in 30 ppm sodium hypochlorite solution, each separated by rinses in sterilized embryo medium. To address this issue, we designed more rigorous embryo disinfection procedures based upon previous studies of surface disinfection designed to rear gnotobiotic zebrafish that are entirely lacking in any host-associated microbes.^8,9^ Gnotobiotic disinfection procedures use antibiotics, povidone–iodine (PVP-I), and sodium hypochlorite to completely eradicate microbes on the surface of the chorion.^8,9^

We generated green fluorescent protein (GFP)-expressing *M. marinum* using isolates from our colony,^10^ and using these GFP-expressing bacilli and zebrafish embryos aged 5–6 h postfertilization, we evaluated the effectiveness of a PVP-I treatment used for gnotobiology disinfection (1000 ppm PVP-I for 2 min) and a sodium hypochlorite treatment used for gnotobiology disinfection (30 ppm sodium hypochlorite for 20 min).

**FIG. 1.**
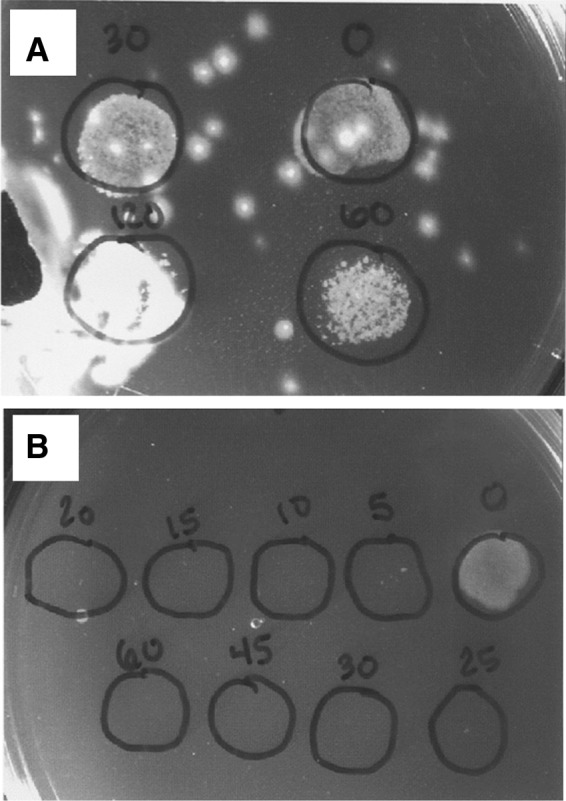
Comparison of fluorescent *Mycobacterium marinum* (5.95 × 10^8^ CFU/mL) after exposure to 1 part per thousand (ppt) PVPI for 2 min **(A)** and 30 ppm sodium hypochlorite solution for 10 min **(B)**. Numerals represent exposure time in seconds **(A)** and minutes **(B)**. Cultures grown on Middlebrook 7H11 media; *circles* indicate where bacteria were spotted onto the plate for testing. Sodium hypochlorite essentially eradicated *M. marinum* after 10 min, whereas PVPI had almost no effect. PVPI, povidone–iodine.

We first tested the effectiveness of the disinfecting agents on *M. marinum* in culture. After these experiments, we focused on using sodium hypochlorite because we observed that it eradicated more *M. marinum* in culture than PVP-I (Fig. 1). We next tested the survival of *M. marinum* when exposed to a series of sodium hypochlorite solutions at various concentrations and durations in culture and, after determining the concentration and duration required to eradicate the bacilli, we then immersed live embryos in a solution containing our GFP-expressing *M. marinum* and tested their survival using the same concentration and duration.

**FIG. 2.**
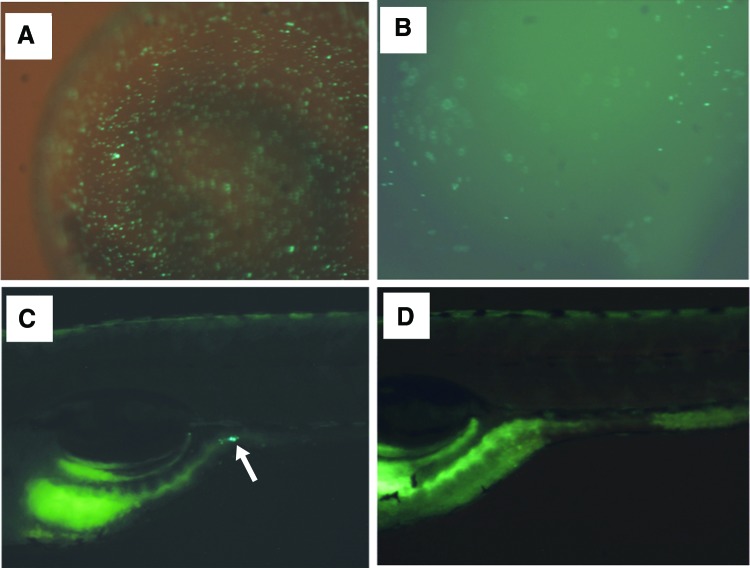
Without surface disinfection, GFP-expressing *M. marinum* were observed on the embryo chorion **(A)** after 24 h and in the larval zebrafish gut **(***arrowhead* in **C)** after 6 days. GFP-expressing mycobacteria were present on the chorion **(B)** after 24 h, but absent in the larval zebrafish gut **(D)** after 6 days when the chorion was surface disinfected using 30 ppm sodium hypochlorite for 10 min. Larvae are positioned anterior to the *left*, dorsal up. GFP, green fluorescent protein. Color images available online at www.liebertpub.com/zeb

We observed that 20 min of exposure to 30 ppm sodium hypochlorite eradicated all *M. marinum*, but most embryos did not survive (not shown). We further observed that GFP-expressing *M. marinum* was only present in the gut of larvae and not in any other area of the fish after 7 days of exposure (not shown). We also observed that although some GFP-expressing *M. marinum* were found on the embryo chorion after 10 min of exposure to 30 ppm sodium hypochlorite, no GFP-expressing *M. marinum* were observed in the gut of the larval zebrafish after 6 days (Fig. 2). We ultimately chose a 30 ppm 10-min exposure for our disinfection procedure (see Methods section) because this provided us with maximum embryo survival and no observed GFP-expressing *M. marinum* in larvae.

**FIG. 3.**
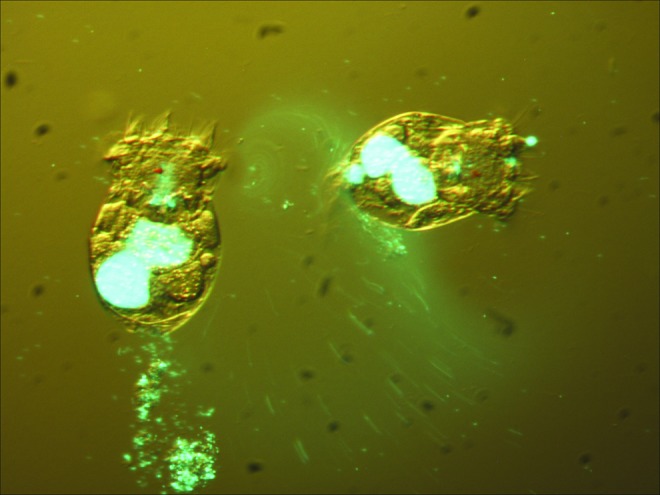
Rotifers (*Brachionus plicatilis*) readily consume GFP-expressing *M. marinum*. Color images available online at www.liebertpub.com/zeb

Through environmental sampling, we discovered we had the pathogenic mycobacteria in our live feed cultures (Table 1). Experiments with the GFP-expressing *M. marinum* revealed that the rotifers in our cultures readily consumed the *M. marinum* (Fig. 3), making these a possible source of infection to our larval zebrafish. To investigate the source of this contamination, we purchased and tested weekly shipments of live rotifers from the vendor and systematically tested, then destroyed our 1-week old cultures. Tests of live rotifers from the vendor showed no mycobacteria (*n* = 24), whereas tests of our 1-week old culture showed two contaminations several weeks apart (Table 4). From these tests, we concluded that accidental transfer of fish water to rotifer cultures, possibly as the result of personnel movements, was the cause of our culture contamination.

**Table 4.** Environmental Sampling for Rotifer Culture Contamination

*Sample type (location)*	*Year-Month*	*Mycobacterium* spp.	*M. chelonae*	*M. marinum*
Biofilm (bucket wall)	2013-06 (*n* = 1)	+	−	+
Biofilm (bucket wall)	2013-07 (*n* = 1)	+	+	+
Biofilm (bucket wall)	2013-08 (*n* = 1)	−	−	−
Live rotifers (vendor-supplied)	2013-08 (*n* = 2)	−	−	−
Live rotifers (laboratory culture)	2013-08 (*n* = 2)	−	−	−
Live rotifers (vendor-supplied)	2013-09 (*n* = 4)	−	−	−
Live rotifers (laboratory culture)	2013-09 (*n* = 4)	−	−	−
Live rotifers (vendor-supplied)	2013-10 (*n* = 5)	−	−	−
Live rotifers (laboratory culture)	2013-10 (*n* = 5)	−	−	−
Live rotifers (vendor-supplied)	2013-11 (*n* = 4)	−	−	−
Live rotifers (laboratory culture)	2013-11 (*n* = 4)	−	−	−
Live rotifers (vendor-supplied)	2013-12 (*n* = 5)	−	−	−
Live rotifers (laboratory culture)	2013-12 (*n* = 5)	−	−	−
Live rotifers (vendor-supplied)	2014-01 (*n* = 4)	−	−	−
Live rotifers (laboratory culture)	2014-01 (*n* = 4)	+	−	+
Biofilm (bucket wall)	2014-01 (*n* = 7)	−	−	−
Biofilm (bucket wall)	2014-08 (*n* = 1)	−	−	−
Biofilm (bucket wall)^a^	2015-05 (*n* = 1)	−	−	−
Biofilm (bucket wall)	2015-10 (*n* = 3)	−	−	−

^a^ Rotifer cultures were moved to a dedicated food room in April, 2015.

+ indicates that this area was positive for mycobacteria at least once.

− indicates that all samples from this area were negative for mycobacteria.

To correct this problem of feed contamination, we directed personnel to perform husbandry tasks in a defined order and specifically to carry out rotifer culture tasks before tasks with zebrafish. We ultimately moved our rotifer cultures to a dedicated food preparation room to avoid any possibility that water contaminated with mycobacteria could enter the live feed cultures, and we continued to instruct personnel to perform rotifer culture maintenance before any zebrafish work.

To more effectively find and remove infected zebrafish, we changed the way we performed daily health checks and rather than combining the health check with our feeding procedure as we had done previously, we dedicated time and personnel to survey facility tanks for infected, moribund, and dead fish. To track infections in the colony, we used brightly colored single-purpose labels to mark specific tanks after removing from them individual zebrafish showing signs of infection. This single-purpose labeling of tanks with infected fish provided more immediate health status overviews of large groups of tanks than our previous method that had used clipboards on the ends of racks to track diseased fish.

Other strategies that we used to reduce the number of fish with infections focused on pathogen spread through water transfer and through fish-to-fish contact. Because incidence of disease increases with age,^11–13^ we included a regular review of fish ages to remove elderly individuals (>600 dpf) from the colony. Before the outbreak, we had a 600-day rule, but no central tracking of the age of colony fish, and removing fish aged over 600 days was not enforced. During the outbreak, in our urgency to remove potentially diseased individuals from our colony, those older zebrafish not useful as broodstock were removed, unless they were used for a specific study such as tumor growth.

We used a custom database (FileMaker Pro, FileMaker, Inc., Santa Clara, CA) to track batches of zebrafish (stocks) and each tank had a label affixed to its front showing information about the zebrafish inside it, including the fertilization date. We posted signs around the facility that allowed all users to easily compare dates and ages and our facility manager and veterinarian helped to enforce the elderly fish rule. Similarly, because of concerns over disease in older fish and because water is an excellent vehicle for transmitting pathogens,^14^ we placed tanks containing juvenile fish above tanks containing older fish on our racks to minimize the spread of the disease to juveniles when water drips or spills occur and water moves from tank to tank.

To further prevent spread of the pathogen through water and infected fish, we discontinued our previous practice of maintaining groups of centrally managed wild-type zebrafish for researchers to use in outcrosses. Previously, we housed some wild-type fish in large shared tanks and we allowed researchers to remove fish from these tanks, use the fish in outcrosses with mutant strains, and then return the wild-type fish for subsequent use. After the outbreak, we distributed smaller groups of wild-type fish for each laboratory to use, and in cases in which a mutant strain was represented in low numbers of colony fish or the strain was known to be immunocompromised, we followed a practice that Murray *et al.* recommended and used in the control of *Pseudoloma neurophilia*,^15^ setting aside a dedicated subset of wild-type fish for outcrosses to the specific strain.

Some strategies that we implemented to eliminate the existence and spread of mycobacteria focused on our equipment sanitization and included a more frequent tank change schedule and modified equipment sanitization procedures. We began using a 3-week schedule instead of a 6-week schedule for housing tank changes because mycobacteria have been found in the biofilm from tank walls,^16^ and the same strains have been identified in both biofilms and fish.^17^ We did not test for biofilm, but rather assumed the reduced time that tanks were exposed to water and zebrafish correspondingly reduced biofilm and algae growth on tank walls. The tank changes were tracked using a posted schedule aligning weeks with rack rows. We used both facility staff and student workers to accomplish the 3-week tank change schedule.

We also changed our equipment sanitization procedures, and we programmed the washer cycles we used to sanitize tanks and other equipment to reach at least 60°C because *M. marinum* was shown to be susceptible to this temperature.^18^

During the outbreak, because we wanted to expose tank surfaces to high temperatures, we discontinued our previous practice of cleaning large glass tanks with fish in them and replaced our large glass tanks with smaller polycarbonate tanks that we found easier to move. Before the outbreak, we housed fish in either 1-gallon polycarbonate tanks or 29-gallon glass tanks.

We sanitized the polycarbonate tanks on a 6-week schedule by transferring the fish to a clean tank, then pretreating the dirty tank for 1 h in a 1.2% sodium hypochlorite bath, followed by thoroughly washing and rinsing the tank in a mechanical washer (Amsco Glassware Washer Model 400 CW). We cleaned the glass tanks with fish in the tank by scrubbing interior surfaces using a pad (3M Scotch-Brite 96-20 General Purpose Scouring Pad, hand-sewn) attached to a long acrylic bar, followed by detritus removal using an acrylic siphon tube.

Only after all the fish in a glass tank were no longer needed and completely removed, did we remove the glass tank from its rack position and thoroughly sanitize it with 1.2% sodium hypochlorite solution, followed by municipal water rinses. Importantly for us, the washer did not have a guarantee on the wash cycle temperatures. It was possible for equipment to pass through the washer without reaching 60°C.

After the outbreak, although not as a response to it, we purchased two new mechanical washers and, after counsel from our washer manufacturers about bleach in the washers, we discontinued the use of sodium hypochlorite in our equipment pretreatment. We changed our cleaning procedure to use brushes (Bar Maid, Pompano Beach, FL) for optional prescrubbing of soiled tank walls, lids, and other tank parts using municipal water. We soaked heavily soiled lids overnight in municipal water with no added detergent before washing. We washed all our polycarbonate tanks in a mechanical washer (410LX; Lynx PG, Wilson, NY) using a cycle programmed with both alkaline (Clout PF) and acid (Urid) detergents (Pharmacal, Waterbury, CT), followed by rinses of nondetergent municipal water heated to at least 60°C and a final rinse using nondetergent municipal water heated to 82°C.

For equipment that would not fit on washer racks designed for tanks, we optionally prescrubbed tank lids and other tank parts using our brushes, then we washed them in a mechanical washer (400XLS; Steris Corp., Mentor, OH) using a cycle programmed with alkaline detergent (Clout PF, Pharmacal, Waterbury, CT), followed by rinses of nondetergent municipal water heated to at least 60°C and a final rinse using pure water heated to 82°C. By using our mechanical washers, both of which were equipped with guarantees for cycle temperatures, and by validating the high heat of the cycles using temperature tapes, we assured ourselves that our equipment was effectively and reliably sanitized.

At all times, before and after the outbreak, we sanitized all equipment used in the cleaning of a tank before subsequent use and we also sterilized nets in an autoclave before their reuse. Our primary concern for the sanitation process was our reliance on sodium hypochlorite as a disinfectant for soiled equipment. Sodium hypochlorite loses its effectiveness over time and in the presence of organics.^19,20^

For equipment used before the outbreak, we washed scrub pads in warm water to remove detritus and algae, then sterilized them in an autoclave; however, we submerged acrylic rods and siphon tubes in a bath of 1.2% sodium hypochlorite for a minimum of 1 h, transferred them to a neutralizing bath of sodium thiosulfate, then returned them for use. We believed that in our facility with our washroom throughput and staffing, by using mechanical washers and validating cycle temperatures using temperature tapes, we could improve the reliable eradication of pathogens on our soiled equipment. Therefore, after the outbreak, we used our washers and our autoclave to sanitize and disinfect our equipment.

**FIG. 4.**
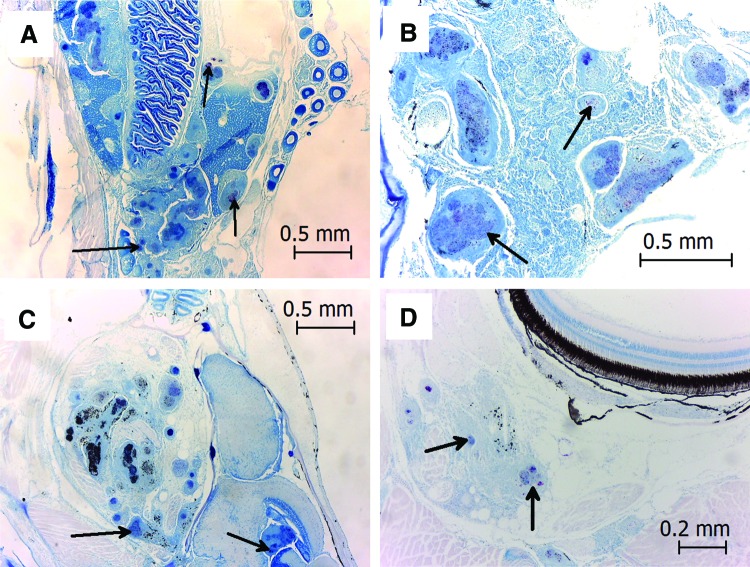
Severe mycobacterial infection in a single adult zebrafish. *Arrows* pointed to acid-fast bacilli that are observed in the coelomic cavity, including ovary **(A)** and kidney **(B)**, but are also observed in the brain **(C)** and choroid **(D)**. Color images available online at www.liebertpub.com/zeb

Our histological examination of sampled zebrafish showed that the mycobacteria were present throughout afflicted zebrafish tissues, including kidney and ovary, as we had previously observed, but during the outbreak, we also observed acid-fast bacilli in the brain as well as the ocular cavity and choroid (Fig. 4), suggesting to us that our zebrafish were particularly susceptible to this strain of *M. marinum.*

Before the outbreak, because of concerns related to water loss in our RAS, during tank changes and embryo collection from breeding cages, dirty tank water and breeding cage spawn water were directed to the return water stream and conserved for the RAS. Given that ulcerative lesions were likely a source of mycobacteria in tank water from infected fish,^21^ and that observed ulcerative lesions may be indicative of systemic mycobacteriosis, we decided we should discard water from housing tanks and spawn water from breeding tanks instead of saving it for our RAS. Therefore, we changed our practice of conserving housing tank water during our tank change procedure and breeding tank water during embryo collection to one of discarding the water.

Last, concerns about transferring the *M. marinum* pathogen to our collaborators during animal transfer resulted in a new process for our zebrafish exports, initiated by our Institutional Official, which mandated a disclosure form and pretransfer dialog between members of our animal welfare program, usually the attending veterinarian, and an authorized official from the receiving institution. Because our group is funded, in part, by Public Health Service (PHS) funds, we are required to use the *Guide for the Care and Use of Laboratory Animals* (the *Guide*) as a basis for our animal welfare program^22^ and, for the transportation of animals, the *Guide* asserts that the veterinarian or the veterinarian's designee should review the health status and other requirements before allowing the shipment of animals to ensure that (in part) effective quarantine is implemented.^23^

The disclosure was designed to provide an opportunity for the receiving institution to review its biosecurity and its ability to protect its personnel and zebrafish colony from the potential import of *M. marinum* along with the zebrafish and is in accord with the *Guide.*

## Methods

### Histology

Zebrafish were euthanized by rapid chilling, then preserved whole in Dietrich's fixative, processed, embedded in paraffin, sectioned sagittally, and stained with hematoxylin and eosin (H&E) or Ziehl-Neelsen's acid-fast by routine methods. Identification of *Mycobacterium* species was performed at the Oregon Veterinary Diagnostic Laboratory (Corvallis, OR), where tissues containing acid-fast bacteria were cored from paraffin blocks and subjected to specific PCR tests targeting the *hsp65* gene in real-time PCR format.

### Environmental sampling

Sterile flocked swabs (Copan FLOQSwabs #520CS01) were used to gather environmental material, for example, biofilm, algal growth, dry feed, live feed, and detritus, then placed in Eppendorf tubes (Eppendorf #022363204), cataloged, and either transferred to a laboratory locally at the University of Oregon (UO) or sent to a commercial diagnostics laboratory (IDEXX BioResearch, Columbia, MO) for mycobacterial testing through PCR.

For work done at UO, DNA was extracted from the environmental samples. Samples were then amplified using the T39 and T13 primers described in Talaat *et al.*^34^ to reveal a 924 bp *Mycobacterium* band. This band was then restriction digested and assayed for *M. marinum*-specific fragments using Apa, which generates fragments of 677, 132, and 115 bps and distinguishes *M. marinum* from *M. chelonae*, and Ban, which does not cut in *M. marinum*, but differentiates *M. marinum* from *M. fortuitum.*

### Preparation of fluorescent *M. marinum*

The vector pTec15, a gift from Lalita Ramakrishnan (Addgene plasmid #30174), encoding Wasabi fluorescent protein under the control of a strong *Mycobacterium* promoter was transformed into *M. marinum* by electroporation as previously described.^10^

### Embryo disinfection

Embryos housed at 28.5°C (±1.0°C) for 5–6 h postfertilization (developed to 50% epiboly) were disinfected by immersion in a solution of 30 ppm laboratory-grade sodium hypochlorite (Fisher Scientific #SS290-1) continuously for 10 min, followed by three rinses in embryo medium. No pH adjustments were made to the sodium hypochlorite disinfection solution. Embryos were grouped into batches of no more than 100 individuals and placed in cylinders with mesh bottoms for easy transfer between disinfection solution and rinse solutions.

### Material toxicity test

Candidate nitrile gloves were tested for toxicity to zebrafish by placing a sample of each glove with fertile zebrafish embryos. After 7 days, zebrafish exposed to glove material were screened for developmental deformities and mortalities compared with a control group.

## Discussion and Recommendations

Our strategies to mitigate the effects of our *M. marinum* outbreak were focused first on protecting our personnel, then on containing the spread of the pathogen among fish within the facility (Table 3). We have seen a decline in confirmed cases of mycobacterial infection caused by *M. marinum* since we implemented these strategies, although because many of them were reasoned and not tested, we cannot prove that the decline is the direct result of all these measures. Nonetheless, we believe other facilities can benefit from our experiences.

### Personnel concerns

Personnel who have direct contact with zebrafish are at risk for zoonotic infection. Many species of mycobacteria are associated with mycobacterial infections of zebrafish^1,2^ and generally considered opportunistic pathogens.^1^ A significant concern with these bacteria that live in biofilms as well as inside the zebrafish^1^ is the risk to personnel exposed to these potentially pathogenic bacteria. All species of mycobacteria that cause infections in laboratory zebrafish are zoonotic pathogens.

In a retrospective study on nontuberculous mycobacteria (NTM)-induced cutaneous infections seen in a hospital dermatology outpatient clinic during a 14-year period, Dodiuk-Gad *et al.* noted that *M. marinum* was the cause of the patient skin infection in a majority of cases.^24^ The same study found that *Mycobacterium chelonae*, *Mycobacterium xenopi*, *Mycobacterium abscessus*, *Mycobacterium gordonae*, and *Mycobacterium fortuitum* were also responsible for some instances of NTM-induced cutaneous infection.^24^

Although hand infections caused by *M. marinum* have been observed in aquarists for decades, Ostland *et al.*^25^ were the first to genetically link the same strain of *M. marinum* in fish and human lesions using DNA fingerprinting. In our facility, we implemented PPE, especially gloves, for our personnel only after we had a confirmed zoonotic infection. We recommend that other facilities consider protecting their personnel by implementing glove use before any zoonotic infection occurs.

Personnel can facilitate the spread of a pathogen. Our experiences with the contamination of our live feed cultures as well as our discovered contamination of a computer keyboard and equipment cabinet handles showed us that our personnel can inadvertently become vectors for pathogens such as mycobacteria. Personnel training with an emphasis on laboratory zebrafish diseases, physical and behavioral signs of disease in fish, and the dangers of fomites and contamination from soiled equipment have become essential components of our training for new personnel. We recommend that other facilities implement training for new personnel on laboratory zebrafish diseases.

### Animal concerns

As we discovered, rotifer cultures, like other live feed cultures, can become contaminated with pathogens^13,26^ because the rotifers can consume a varied diet of microalgae, bacteria, flagellates, and small ciliates.^27^ Live feed for first-feeding larval zebrafish is common in laboratories using zebrafish as a model organism^13,28–30^ and, consistent with this practice, our facility feeds live rotifers to first-feeding larvae and our live rotifer cultures are an essential part of our rearing procedure. Moreover, it has been shown that another live feed type, paramecia (*Paramecium caudatum*), not only concentrate mycobacteria, but also bacteria within the paramecia are actually more infectious than their counterparts grown in artificial media.^26^

Given our experiences with our live culture contamination and our evidence that rotifers could potentially transmit mycobacteria to larvae, we recommend careful attention to the placement of live rotifer cultures away from zebrafish and water used with zebrafish to avoid rotifer culture contamination by disease-causing bacteria.

Our animal transfer export disclosure statement formalized the process of sending our zebrafish to other institutions and, in a beneficial way, provided other facilities and institutions the opportunity to perform a risk analysis before the receipt of any fish. Implementing this disclosure and having conversations between our attending veterinarian and authorized officials from other institutions revealed to us that many institutions either do not have a sufficiently robust health program to know whether fish within their facility carry any serious pathogens or they are unwilling to disclose information about pathogens. Until zebrafish facilities implement a common disclosure program, all facilities need to remain vigilant whenever they import fish.

We recommend using a quarantine rack or quarantine facility away from main housing and we further recommend a period of observation for all imported zebrafish before their use. We wait for a minimum of 3 weeks before handling and breeding newly imported zebrafish, but we prefer to wait for 5 weeks before using them because we have seen signs such as ulcerative lesions appear as late as 4 weeks after import.

Our response to the *M. marinum* outbreak in our zebrafish colony was to first protect our personnel, then to investigate the prevalence and spread of the mycobacteria in our facility, and finally to change many of our practices to mitigate the outbreak. Our discoveries showed us that personnel, zebrafish, and water exposed to zebrafish were sources of contamination.

In our disinfection studies, we found that exposing zebrafish embryos aged 5–6 h postfertilization to 30 ppm sodium hypochlorite for 10 min prevented any occurrence of GPF-expressing *M. marinum* in the 7-day-old larvae that later developed. We observed GFP-expressing *M. marinum* in the gastrointestinal tract of 7-day-old larvae that developed from embryos that were not disinfected. Exposure of sodium hypochlorite for 20 min killed both the bacilli and the fish. This 30 ppm sodium hypochlorite exposure is consistent with previous observations that fish can tolerate concentrations as high as 100 ppm or more, but time of exposure is also important.^31^

We did not find povidone–iodine to be effective in eliminating *M. marinum*, even at 1000 ppm (Fig. 1). This is in contrast to another study that found 25 ppm povidone–iodine for 5 min to be very effective in killing several species of mycobacteria.^32^ The authors emphasized the importance of preparing iodine solutions fresh, as after 24 h, a diluted solution has an almost 10-fold decrease in iodine.^32^ Iodine has also been shown to be effective in killing *M. marinum* and safe for embryos with 2-min exposures at 12.5–25 ppm.^33^ Ultimately, we used sodium hypochlorite for embryo disinfection, but there may be other effective alternatives.

By devising strategies to combat the pathogenic mycobacteria and by refining our procedures, we were able to minimize its impact on our research program. We believe this case report provides practical methods that can be used in other facilities to mitigate similar outbreaks.
